# Supplementary material for: Influence of Glucocorticoids on Cellular Senescence Hallmarks in Osteoarthritic Fibroblast-like Synoviocytes
Source: J Clin Med. 2021 Nov 16;10(22):5331. doi: 10.3390/jcm10225331 (PMC8617749; doi:10.3390/jcm10225331)
Supplement: Supplementary file 1 [file jcm-10-05331-s001.zip › jcm-1392361-supplementary.pdf]

Journal of
Clinical Medicine

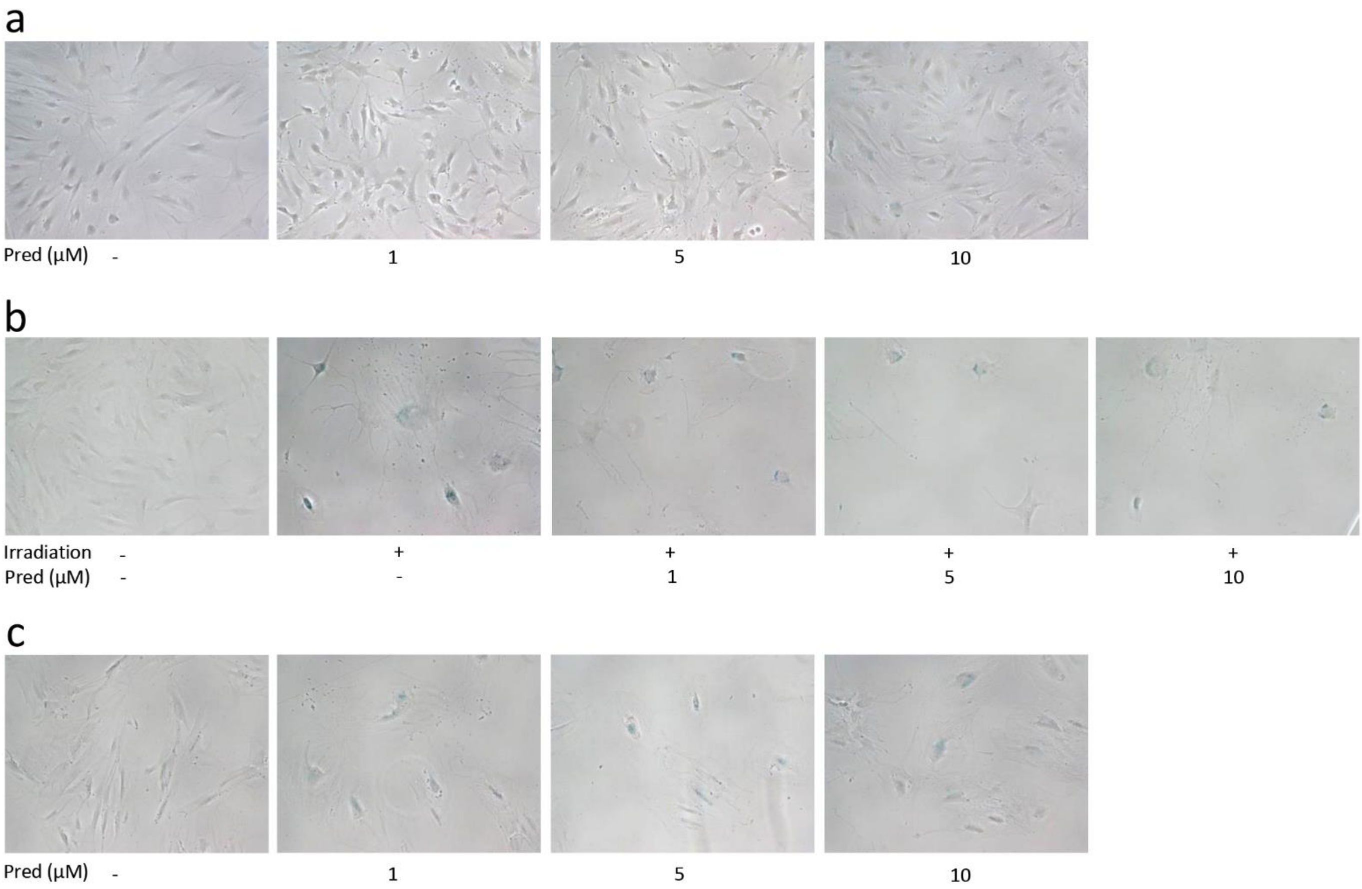

Figure S1. Representative example of β-galactosidase staining. (**a**) Effect of prednisolone on β-galactosidase staining in osteoarthritic fibroblast-like synoviocytes (cells recovering after 8 days). (**b**) Effect of prednisolone on β-galactosidase staining in irradiated osteoarthritic fibroblast-like synoviocytes (cells recovering after 8 days). (**c**) Effect of prednisolone on β-galactosidase staining in mesenchymal stem cells (cells recovering after 8 days).

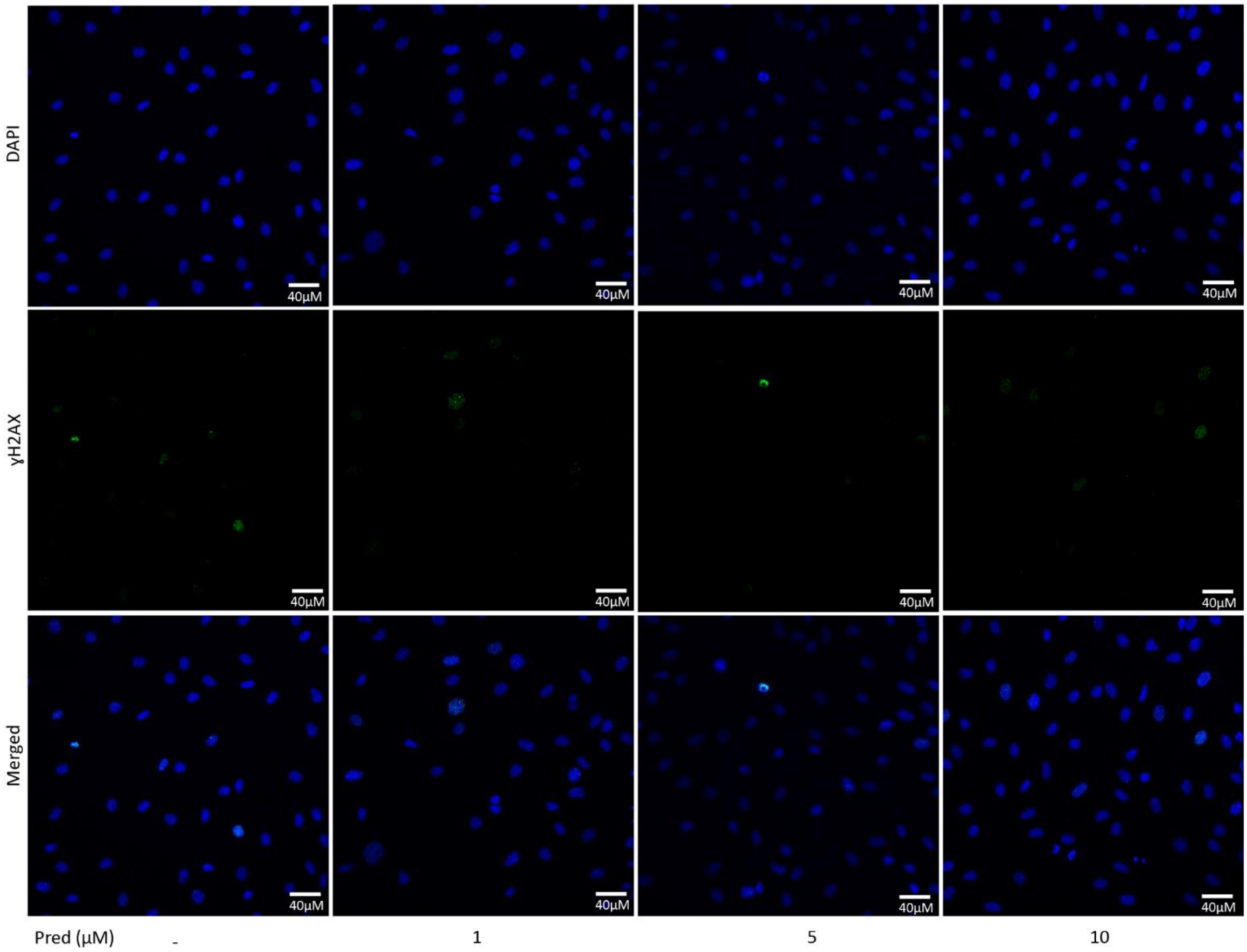

Figure S2. Influence of prednisolone on DNA damage in osteoarthritic fibroblast-like synoviocytes (cells recovering after 8 days). Representative pictures of DNA damage with γH2AX staining.

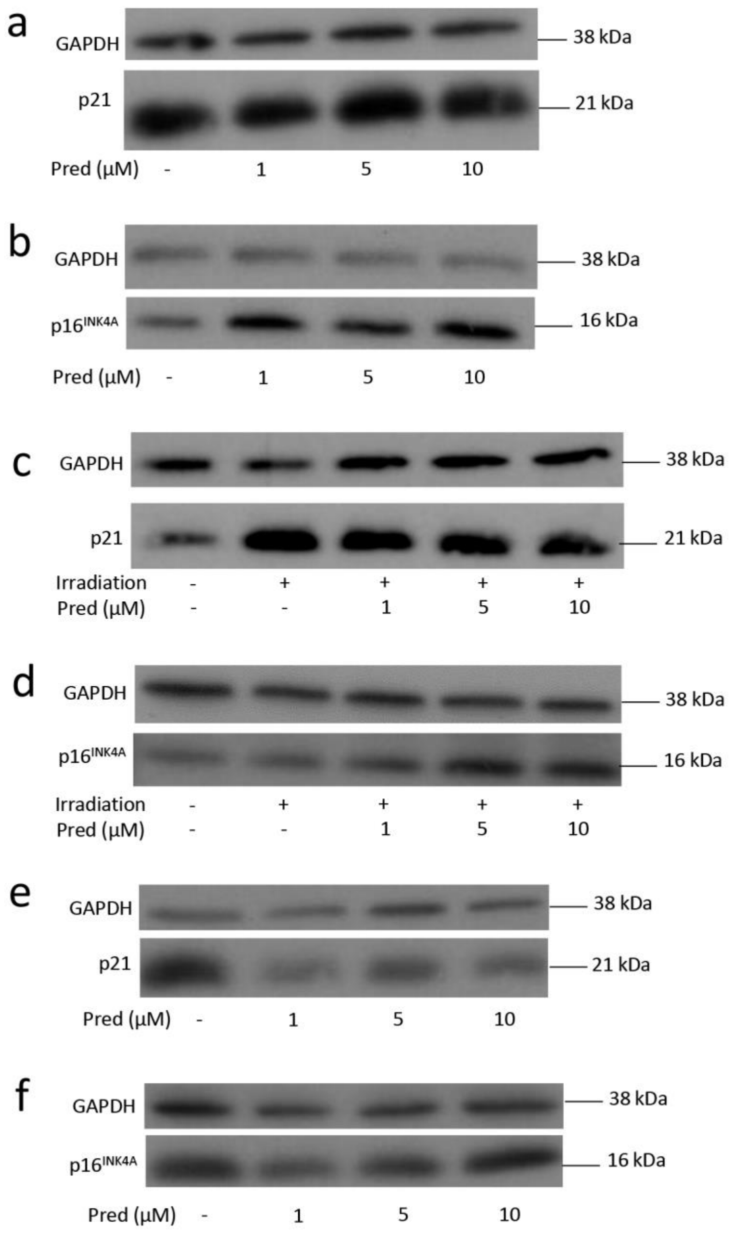

Figure S3. Representative example of Western blotting. (**a**) p21 protein expression in osteoarthritic fibroblast-like synoviocytes (cells recovering after 8 days). (**b**) $p16^{INK4A}$ protein expression in osteoarthritic fibroblast-like synoviocytes (cells recovering after 8 days). (**c**) p21 protein expression in irradiated osteoarthritic fibroblast-like synoviocytes (cells recovering after 8 days). (**d**) $p16^{INK4A}$ protein expression in irradiated osteoarthritic fibroblast-like synoviocytes (cells recovering after 8 days). (**e**) p21 protein expression in mesenchymal stem cells (cells recovering after 8 days). (**f**) $p16^{INK4A}$ protein expression in mesenchymal stem cells (cells recovering after 8 days).

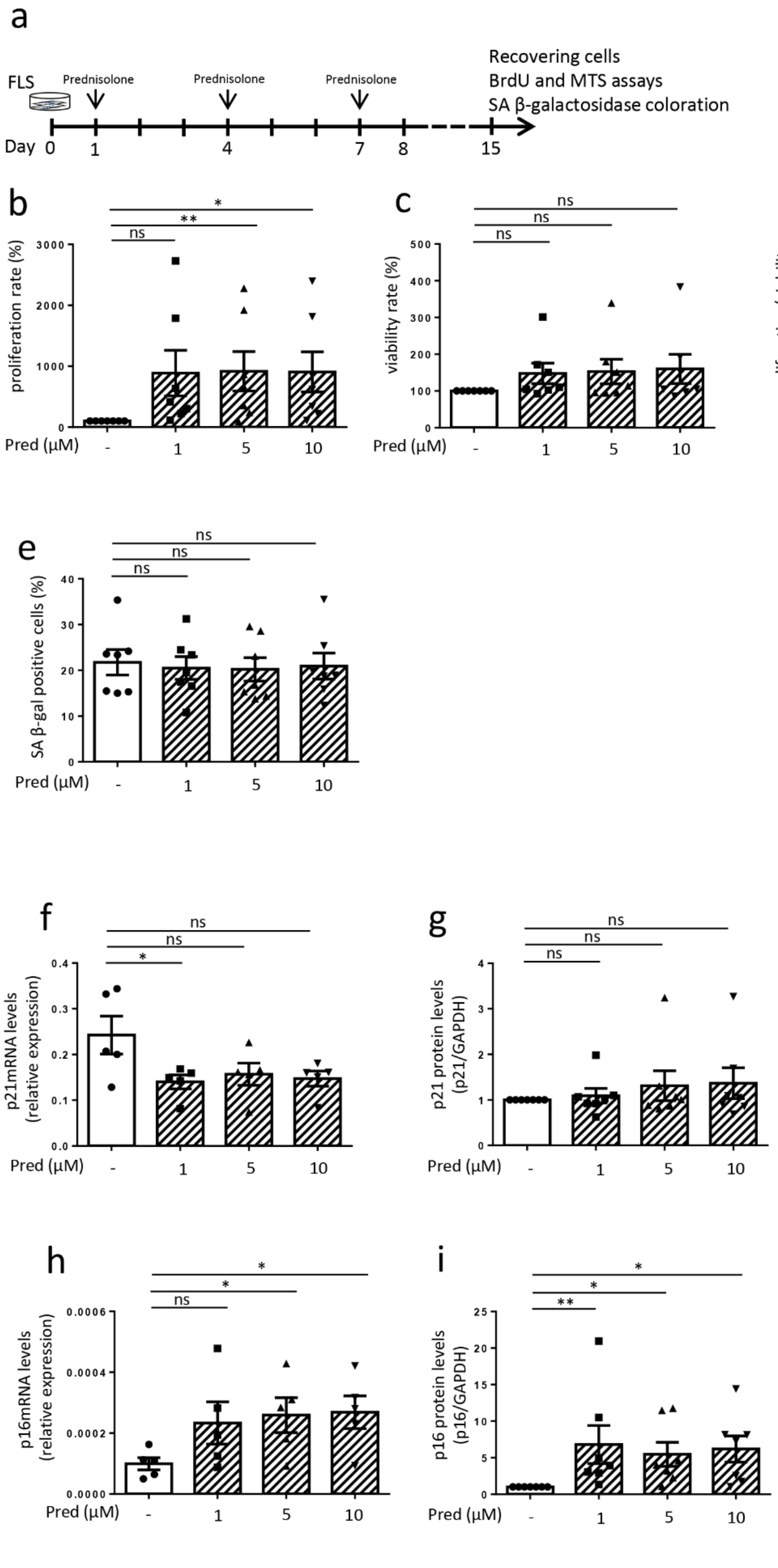

Figure S4. Influence of prednisolone on senescence hallmarks in osteoarthritic fibroblast-like synoviocytes (cells recovering after 15 days). (**a**) Experimental design. (**b**) Proliferation rate analysis (BrdU incorporation) (n=7). (**c**) Viability rate analysis (MTS analysis) (n=7). (**d**) Proliferation/viability ratio (n=7). (**e**) β-galactosidase staining (n=7). (**f-g**) p21 mRNA expression by RT-qPCR (data expressed as 2^-ΔCt, n=5) and protein expression by western blotting (levels reported on

GAPDH, n=7). (**h-i**) p16^{INK4A} mRNA expression by RT-qPCR (data expressed as 2^-ΔCt, n=5) and protein expression by western blotting (levels reported on GAPDH, n=7). Results are expressed as mean ± SEM; * $p < 0.05$, ** $p < 0.01$, ns = not significant.

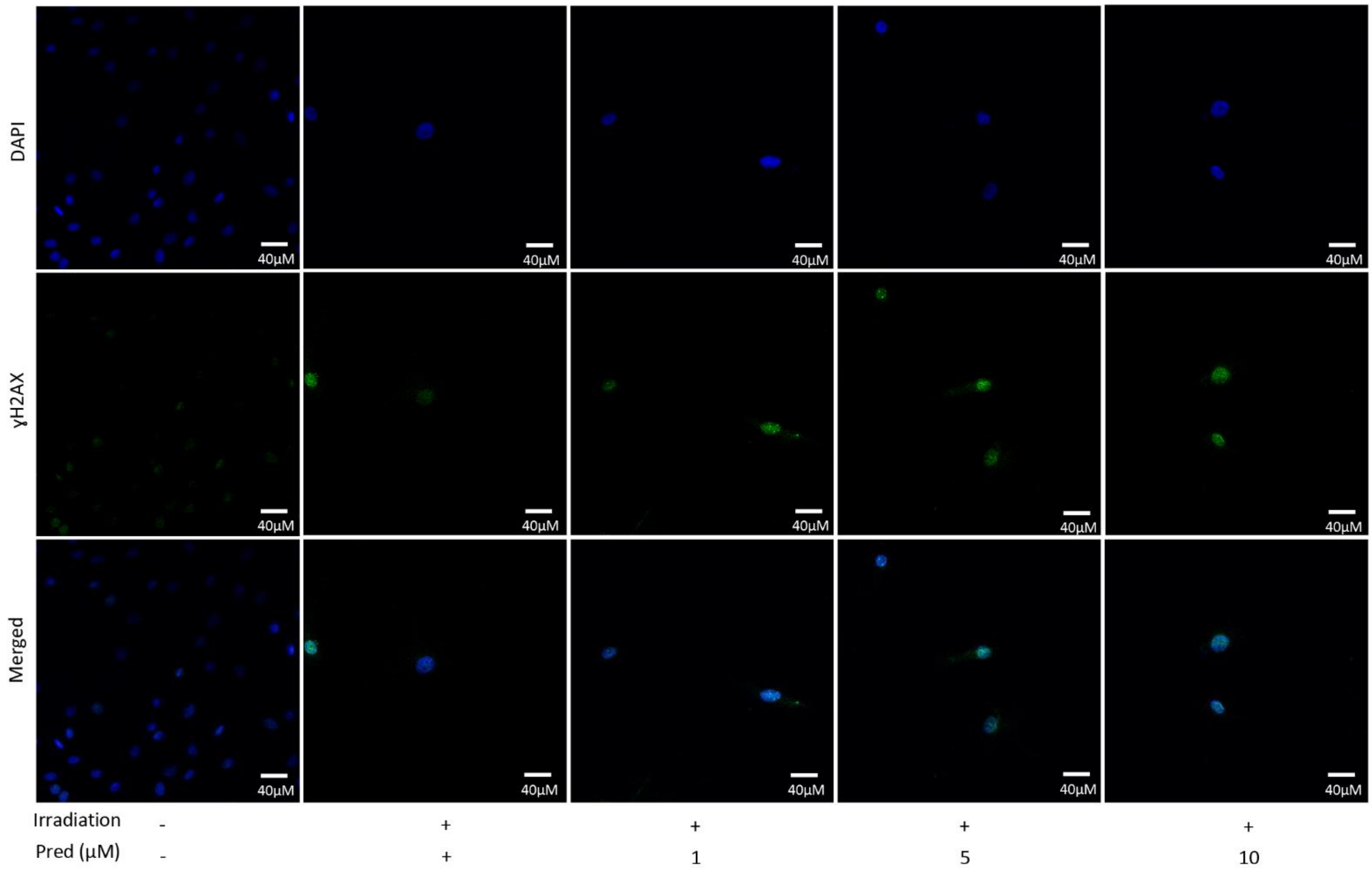

Figure S5. Influence of prednisolone on DNA damage in irradiated osteoarthritic fibroblast-like synoviocytes (cells recovering after 8 days). Representative pictures of DNA damage with ɣH2AX staining.

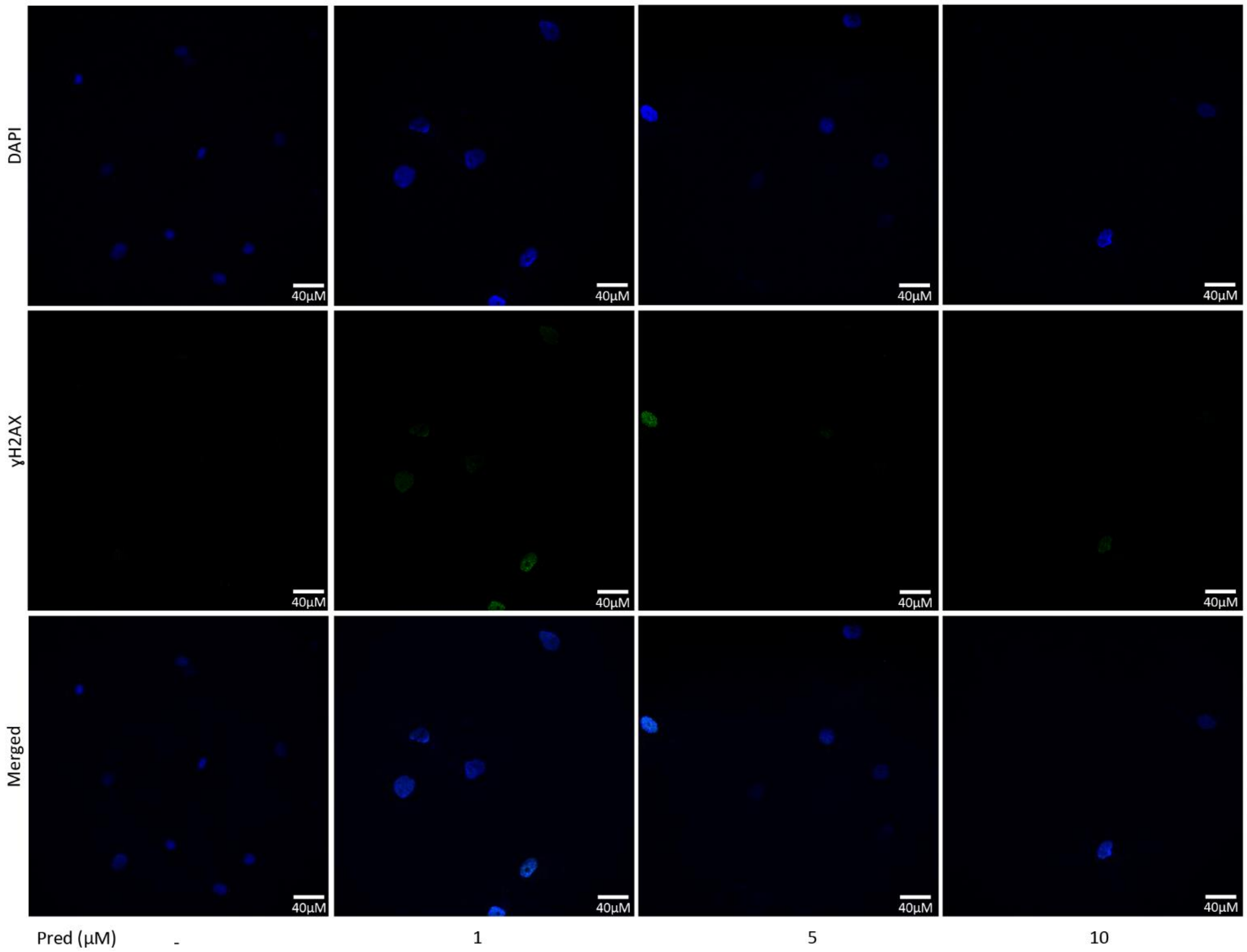

Figure S6. Influence of prednisolone on DNA damage in mesenchymal stem cells. Representative pictures of DNA damage with ɣH2AX staining.
